# Acceptance and effectiveness for learning of a simulation manikin for suprapubic aspiration in toddlers constructed with simple means

**DOI:** 10.1186/s13104-015-1536-7

**Published:** 2015-10-09

**Authors:** Hans Martin Bosse, Alice Martin, Kerstin Ling, Suzan Memili, Silvan Patalong, Veronika Rings, Elisabeth Dorothea Jasper, Katharina Luczak, Svenja Liesenjohann, Alix Witsch, Carolin Wengel

**Affiliations:** Clinic for General Pediatrics, Neonatology and Pediatric Cardiology, University Clinic Düsseldorf (UKD), Heinrich-Heine-University, Moorenstr. 5, 40225 Düsseldorf, Germany; Westphalian Child Centre, Klinikum Dortmund, Beurhausstr. 40, 44137 Dortmund, Germany; Alexianer Hospital Köln, Kölner Str. 64, 51149 Cologne, Germany

**Keywords:** Undergraduate medical education, Clinical skill, Motor skill, Suprapubic aspiration, Puncture, Anatomic model

## Abstract

**Background:**

Skills trainings are increasing in popularity in undergraduate medical education enhancing clinical competencies and motivation for clinical practice. A suprapubic aspiration (SPA) is the gold standard to obtain urine from toddlers and young infants with fever and unclear focus to prove an urinary tract infection.

**Methods:**

In a blended-learning scenario with virtual patients and skills lab training students were trained for a SPA. Currently, no toddler simulation manikin for SPA is available on the market so we constructed one with simple means. Students’ acceptance and their view on relevant aspects of the manikin for learning effectiveness were assessed.

**Results:**

With an expenditure regarding work of 3½ h and material costs of 188.12 Euro we were able to construct a paediatric manikin for suprapubic bladder punction using a cheap basic life support manikin. N = 56 students rated their learning success with the manikin as high (77.2 ± 21.6; mean and standard deviation; visual analogue scales from 100 = totally agree to 0 = don’t agree at all). The model was rated as useful for training (84.2 ± 17.2) and realistic (62.1 ± 23.5). Important factors for students’ learning success were (in descending order) that “urine” could be aspirated (81.4 ± 19.5), the feel of the needle inserted in the manikin (71.5 ± 23.2), and—notably less important—the outer appearance in general (40.3 ± 24.6).

**Conclusions:**

We present a construction of a paediatric manikin for suprapubic aspiration with simple means for a realistic learning scenario with high learning success.

## Background

Simulation-based medical education (SBME) in skills laboratories is increasing in popularity as a methodological teaching approach in medical education worldwide. SBME provides a protective environment [1] that offers students to practice procedures in a simulated setting using manikins, standardized patients, or each other prior to performing procedural skills on real patients [2–4] with the intention to enhance clinical competencies and motivation for clinical practice. SBME has been shown to improve procedural skills both in novices and experts [5–8] when assessed by simulator performance and immediately post-training [3, 9, 10]. There is some evidence that it positively influences the outcome for clinical settings [11]. It seems irrelevant whether trained peer tutors or experienced faculty staff deliver feedback [12–14]. Issenberg et al. defined criteria for an effective implementation of SBME as validity of the simulated scenarios, deliberate practice, feedback and express curriculum integration [9]. Nonetheless little is known about differential impact of varying instructional methods influencing effectivity of SBME for future clinical practice.

### Suprapubic aspiration (SPA) in toddlers and infants

Unexplained fever of 38 °C or higher is common in infants and children, accounting for almost half of all initial visits of infants and children with fever [15]. It is recommended that this subgroup should have an urine sample tested after 24 h at the latest to rule out a urinary tract infection (UTI) [16]. A clean catch urine sample is the recommended method for urine collection, but is associated with contamination and thus false positive results. Since it is important not to misdiagnose a contaminated urine sample as a true UTI or if a clean catch urine is unobtainable technically or without significant delay—as often the case in infants—a urine specimen should be obtained through invasive methods as urethral catheter samples or suprapubic aspiration (SPA; Clinical Practice Guideline of the American Academy of Pediatrics [17]; [14, 18, 19]). Predictive values of both methods are very high and their complication rates comparably low [20] so there is no clear recommendation for either method [21]. Clinical factors to take into account are age, the size of the baby, other co-morbidities and potentially parental preference [21].Urethral catheter samples are more likely to be contaminated than samples obtained by SPA, whereas SPA requires a full bladder [20, 21].

### Study goals

SPA is an important measure in caring for infants with unexplained fever. There are no good data on how well clinicians perform SPA in general, how many complications arise through SPA, and thus how much need there is for training SPA. We assume a strong need for a systematic training in SPA. Our undergraduate skills training aims at improving technical competencies of students in SPA as well as at lowering students’ restraining threshold to perform a SPA. We developed a blended-learning scenario with virtual patients and skills lab training students for training SPA. Unfortunately, no simulation manikin for SPA in toddlers is available on the market so we needed to construct one with as simple means as possible.

Students’ acceptance and their view on relevant aspects of the manikin for learning effectiveness were assessed.

## Methods

### Setting and participants

Paediatric skills laboratory training is an integral part of the medical curriculum of our faculty for 5th year students at our Faculty Training Centre for Medical Competencies (http://www.trainingszentrum.hhu.de/). In a short introductory seminar we communicated that the procedures within the training are to be performed just as in clinical reality: students were required to perform seriously and under aseptic conditions, to talk to the simulated patient or parents, and reflect the potential impression they make on both. In our blended-learning scenario for training SPA students prepared for the skills laboratory training with virtual patients as described earlier [22] with short video clips focusing on the procedure being helpful from students’ perspective.

N = 61 5th year medical students were trained in four consecutive weeks in winter term 2013/14. Students gave informed consent for participation prior to the study and could opt out not to participate—explicitly without potential disadvantages for their course and/or their concluding examination. Students were trained in pairs of two by peer tutors, both taking (a) the active role performing a SPA and (b) providing feedback guided by a checklist [22]. No general instruction was given at the beginning of the skills laboratory training, so students spent the entire time of their training with repetitive, supervised practice with feedback.

### Student peer tutors

N = 7 medical students from their 3rd–5th year served as trained peer tutors. Peer tutors were trained by experienced clinicians in providing intermittent feedback and received continuous coaching.

### Construction of the manikin

For the construction of our manikin we used the resuscitation trainer Baby Anne^®^ of Laerdal Medical GmbH (Puchheim, Germany). Our orthopaedic technician planned and constructed the manikin with the available material and tools of their laboratory (Koppetsch, Duesseldorf, Germany, http://www.koppetsch.de/).

### Analysis

We calculated the total time for planning and constructing the manikin (in man hours), as well as the expenditure for materials (in Euro).

We reviewed the students’ rating of (a) self-assessment of learning achievement, (b) the suitability and (c) realism of the manikin as well as d) main factors for learning success (ratings on visual analogue scales from 0 = disagree to 100 = fully agree). Values are depicted as mean and standard deviation.

### Ethical approval

Data were collected within the regular, voluntary evaluation process of the Medical Faculty of Düsseldorf, Germany. In light of the described study design, the Ethics Committee of the Medical Faculty of Düsseldorf waived requirements for an ethical approval procedure.

## Results

### Construction of the manikin

The expenditure of time for planning was half an hour, and 3 h for construction in cooperation with our orthopaedic technician (see Table 1). Expenditure for materials was 188.12 Euro (see Table 2). The torso was cut open and margins were reinforced with bias tape. The resulting opening was designed to slightly overlap the inserted pad serving as abdominal wall (i.e. Diabetic Injection Training Pad 7070, Erler-Zimmer GmbH & Co. KG, Lauf, Germany). A bow resembling the pelvic symphysis and a hemispheric inner shell was constructed.

**Table 1:** Expenditure of time for planning and construction of the mannequin

Materials	Time (man hours)
Planning	0.5
Constructing the bow	0.58
Constructing the inner shell	1
Seams	1
Adjusting the materials	0.42
Total	3.5

Expenditure of time for planning and construction of the mannequin is calculated in man hours

**Table 2:** Expenditure for materials

Materials	Price (Euro)
Baby Anne manikin (from 4-pack), article no. 050010	112.90
Pad (as spare part)	45.22
Cotton, thread, fabric (approximation)	10.00
Bow (approximation)	10.00
Inner shell (approximation)	10.00
Total	188.12

Materials and costs (in Euro) for construction and assembling of the manikin are listed

### Assembling the manikin for training sessions

**Fig. 1:**
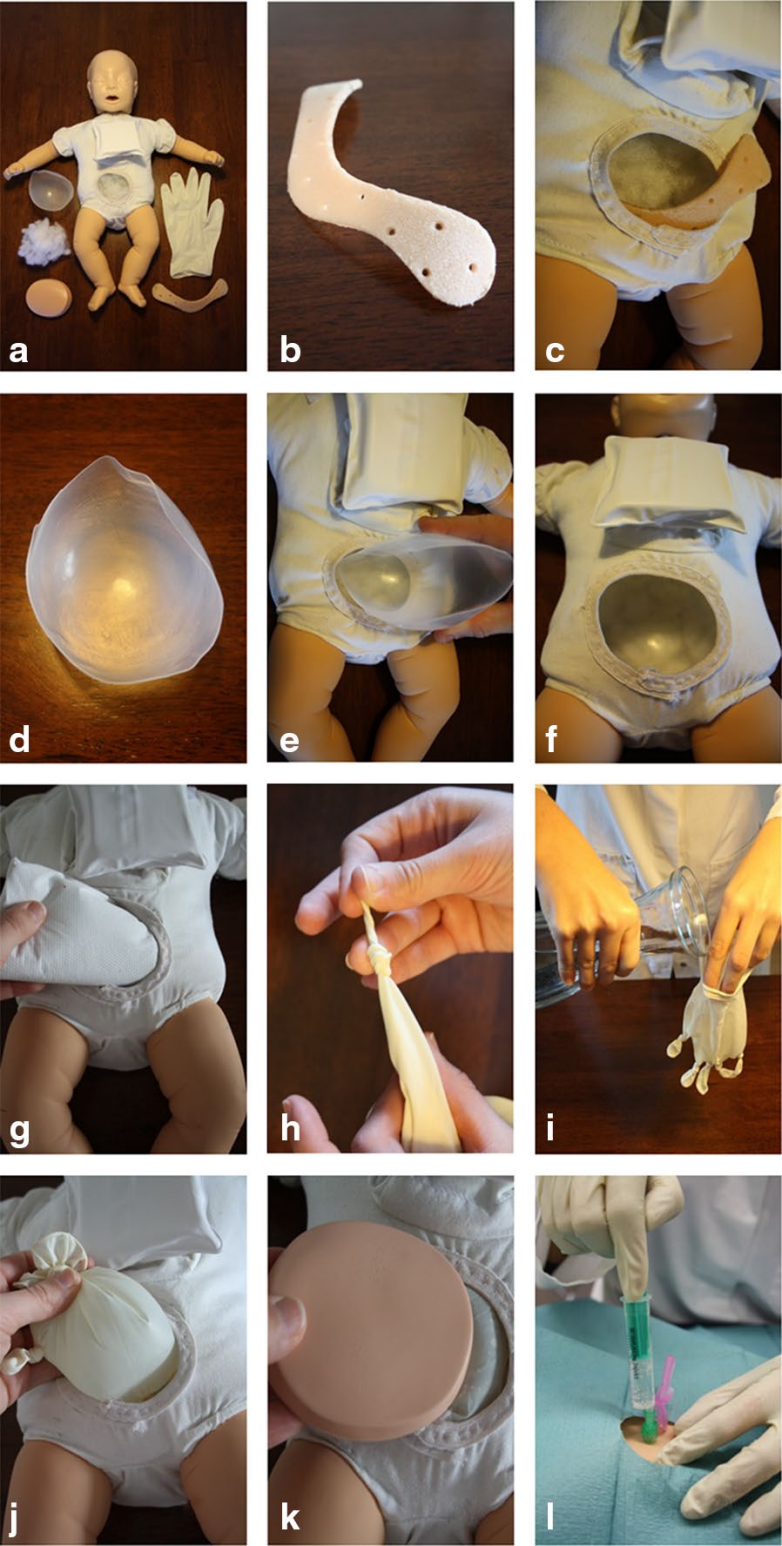
Material, assembly and utilization of the manikin. Material (**a**), construction and assembly (**b**–**k**) as well as utilization (**l**) of the manikin are depicted

The materials for assembling the manikin are depicted in Fig. 1a. A bow resembling the pelvic symphysis (Fig. 1b) is inserted into the torso (Fig. 1c). This will be the landmark to determine the position of the needle for aspiration. A hemispheric inner shell (Fig. 1d) is inserted to stabilize the torso during puncture and to catch leakage from the simulated bladder (Fig. 1e, f). Paper towels serve as padding (Fig. 1g) and allow a smooth and superficial fit of the simulated bladder under the pad.

Two medical disposable gloves serve as simulated bladder. In one glove, all fingers are knotted as shown and it is filled with approximately 50 mL water resembling the volume of a full bladder in the age group relating to the manikins size (Fig. 1h, i). A second glove is pulled over the first one to seal the filled glove. This simulated bladder is put inside the inner shell (Fig. 1j) and covered with a pad (Fig. 1k) which tolerates multiple punctures and may be readily exchanged. The simulated bladder “survives” approximately 6–10 punctures.

### Learning success

Of N = 61 trained students N = 56 completed the questionnaires (return rate 91.8 %). Students rate their learning success as high. The constructed manikin was rated as suitable and realistic. Factors for students’ learning success were (in descending order) to be able to aspirate “urine”, the feel to inserting the needle, and to a much lesser extent the appearance of the model in general (see Table 3).

**Table 3:** Learning success with the mannequin

Item	Mean*	Standard deviation
My learning achievement was high	77.2	±21.6
The model is suitable for training	84.2	±17.2
The model is realistic	62.1	±23.5
Particularly important for my learning achievement in training with the model was …		
… to be able to aspirate “urine”	81.4	±19.5
… the feel to inserting the needle	71.5	±23.2
… the appearance of the model in general	40.3	±24.6

* Assessment with visual analogue scale, from 0 = disagree to 100 = fully agree

## Discussion

Suprapubic aspiration (SPA) is the gold standard to diagnose or rule out a urinary tract infection when a clean catch urine sample is unobtainable technically or without significant delay. It offers little complications in in- or outpatient settings [16, 23] if physicians are proficient, but currently, there are no suitable manikins on the market to train on. We describe and assess a simulation manikin for SPA constructed with simple means. It is a readily accepted, suitable and effective instrument for training SPA and cheap to produce. Students generally view their training on the use on the model as effective. Maintenance and replacing spare parts are easy and quickly done. The acceptance of tutors was high as well (unpublished results of our study group).

Students rated their learning achievement in the training on the manikin as high. We attribute this to the three main factors that Issenberg et al. identify for successful skills training, and which we implemented: providing intermittent feedback, repetitive, active practice and a definite integration into our curriculum [9]. We also assume that preparation with virtual patients (VP) improved students’ performance [22]. An additional point might have been our focus in both VP and in the training on a smooth, automated sequence with a clear goal, on balancing perceived challenge and skills, and providing as much freedom from distractions not immediately related to the process as possible [24].

The key issue from students’ perspective regarding their learning achievement was to be able to aspirate “urine”. This is in line with findings of Wulf et al. that an external focus facilitates automaticity in motor control and promotes movement efficiency [25]. There is no data on whether such a visible “success” as in our simulation scenario is perceived as additional reward or motivation—which both amplify learning processes [26]—or just is surprising.

Another important contribution to students’ learning success was the “feel” while inserting the needle into the manikin. In simple motor skills an immediate sensory-motor feedback may have a comparable effect as a tutor feedback [27]. It remains speculative whether beyond an immediate motoric feedback (merely indicating as appropriate or error) the sensory *feel* during a motoric process is of importance for training (as our participants state). The contribution of an intermediate feedback of tutors (as provided in the study) is unquestionably very high to a successful skills training [28–31]. All the more it is interesting, how much value students attribute to this immediate sensory-motoric feedback.

The students rate the appearance of the model in general less important for their learning success. It is unquestioned that a certain degree of realism and realistic landmarks are essential; virtual reality enhanced manikins may foster the feeling of immersion and realism of the simulation [32]. Thus, manufacturers take great care for realistic appearance of their manikins. But it is unclear which aspects of such “realistic” appearance are significant for improving training success or intended transfer to “real” clinical settings. Further studies should assess which aspects of a training scenario are responsible for creating an image of a human counterpart, and how these may foster soft skills as developing empathy, sensitiveness or feeling responsible. We find students themselves voicing strong attentiveness while inserting the needle into our realistic manikin (unpublished results)—maybe more than if the torso employed was less realistic.

## Limitations

Our data highlight the students’ perspective on their learning with the tool but we don’t provide data from a blinded controlled study design or a pre- and post-assessment. Repetitive deliberate practice with thoughtful feedback as performed in our training is essential for the success of simulation based learning [5] and thus is a potent confounder for the first three items assessing students’ perspective on their learning success and on the manikin as such. Regarding the learning success the correlation between self-assessed efficacy and superior objective performance measures is called into doubt in the literature [33] but higher self-efficacy in skills training results in a more rigorous demand for supervision during the performance of skills in future practice environments [34, 35].

## Conclusions

We present the construction of a paediatric manikin for suprapubic aspiration with simple means for a realistic learning scenario with high learning success from the students’ perspective. We encourage other skills trainers to construct manikins accordingly.

## Abbreviations

SBME: simulation-based medical education
SPA: suprapubic aspiration
UTI: urinary tract infection
